# The β-latch structural element of the SufS cysteine desulfurase mediates active site accessibility and SufE transpersulfurase positioning

**DOI:** 10.1016/j.jbc.2023.102966

**Published:** 2023-02-01

**Authors:** Rajleen K. Gogar, Franki Carroll, Juliana V. Conte, Mohamed Nasef, Jack A. Dunkle, Patrick A. Frantom

**Affiliations:** Department of Chemistry & Biochemistry, The University of Alabama, Tuscaloosa, Alabama, USA

**Keywords:** type II cysteine desulfurase, SufS, suf Fe-S cluster assembly pathway, pyridoxal-5′-phosphate, BME, β-mercaptoethanol, K_D_, dissociation constant, MOPS, 3-(N-morpholino)propanesulfonic acid, NDA, naphthalene 2,3-dicarboxaldehyde, PLP, pyridoxal-5′-phosphate, PDB, Protein Data Bank, TCEP, tris (2-carboxyethyl)phosphine

## Abstract

Under oxidative stress and iron starvation conditions, *Escherichia coli* uses the Suf pathway to assemble iron-sulfur clusters. The Suf pathway mobilizes sulfur via SufS, a type II cysteine desulfurase. SufS is a pyridoxal-5′-phosphate–dependent enzyme that uses cysteine to generate alanine and an active-site persulfide (C_364_-S-S^-^). The SufS persulfide is protected from external oxidants/reductants and requires the transpersulfurase, SufE, to accept the persulfide to complete the SufS catalytic cycle. Recent reports on SufS identified a conserved "β-latch” structural element that includes the α_6_ helix, a glycine-rich loop, a β-hairpin, and a *cis*-proline residue. To identify a functional role for the β-latch, we used site-directed mutagenesis to obtain the N99D and N99A SufS variants. N99 is a conserved residue that connects the α_6_ helix to the backbone of the glycine-rich loop via hydrogen bonds. Our x-ray crystal structures for N99A and N99D SufS show a distorted beta-hairpin and glycine-rich loop, respectively, along with changes in the dimer geometry. The structural disruption of the N99 variants allowed the external reductant TCEP to react with the active-site C364-persulfide intermediate to complete the SufS catalytic cycle in the absence of SufE. The substitutions also appear to disrupt formation of a high-affinity, close approach SufS–SufE complex as measured with fluorescence polarization. Collectively, these findings demonstrate that the β-latch does not affect the chemistry of persulfide formation but does protect it from undesired reductants. The data also indicate the β-latch plays an unexpected role in forming a close approach SufS–SufE complex to promote persulfide transfer.

Fe-S clusters are ubiquitous enzyme cofactors involved in myriad biological pathways across all three domains of life. Owing to the reactive nature of the iron and sulfur building blocks, Fe-S clusters are assembled by highly choreographed multiprotein pathways. These pathways exhibit three conserved steps: (1) sulfur mobilization from cysteine, (2) assembly of the nascent Fe-S cluster on a scaffold complex, and (3) trafficking of the cluster to downstream apoproteins. Two major pathways exist for Fe-S cluster biogenesis, the iron-sulfur cluster (Isc) pathway (1, 2), which occurs in all three domains of life, and the sulfur formation (Suf) pathway (3) which occurs in plants and prokaryotes. *Escherichia coli* contains an example of both the Isc pathway, which is the primary source of Fe-S clusters under normal conditions, and the Suf pathway, which is activated under conditions that disrupt Fe-S metabolism, such as oxidative stress and iron starvation.

The first step in both *E. coli* pathways is the mobilization of sulfur from cysteine by a pyridoxal-5′-phosphate (PLP)-dependent cysteine desulfurase (4). These enzymes catalyze the formation of alanine and a covalent persulfide intermediate on an active-site cysteine (-Cys-S-S^-^) on an S-transfer loop. The persulfide is then transferred to the next protein in the assembly pathway. In the *E. coli* Isc pathway, IscS acts as the cysteine desulfurase and transfers the persulfide directly to the IscU scaffold protein. This is thought to be accomplished by movement of a flexible S-transfer loop containing the active-site cysteine. In the *E. coli* Suf pathway, SufS is the cysteine desulfurase. The active-site cysteine of SufS (C364) is located on a short, inflexible S-transfer loop buried ∼10 Å below the surface of the active-site cleft. Owing to the difference in active-site architecture, SufS requires the accessory protein, SufE, to act as a transpersulfurase, which transfers the SufS-C364-S-S^-^ to SufE-C51-S-S^-^ (Fig. 1) (5). *In vivo*, SufE is regenerated by transferring the persulfide to SufB-C254 in the SufBC_2_D Fe-S cluster assembly complex. For *in vitro* activity, tris(2-carboxyethyl)-phosphine hydrochloride (TCEP) can reduce the SufE persulfide to generate SH_2_. It has been shown that the SufS persulfide is protected from external reductants (6), including TCEP and DTT, and the SufS/SufE system is functional in the presence of hydrogen peroxide (7). All of these characteristics are consistent with the biological function of the Suf system under conditions of oxidative stress.

**Figure 1:**
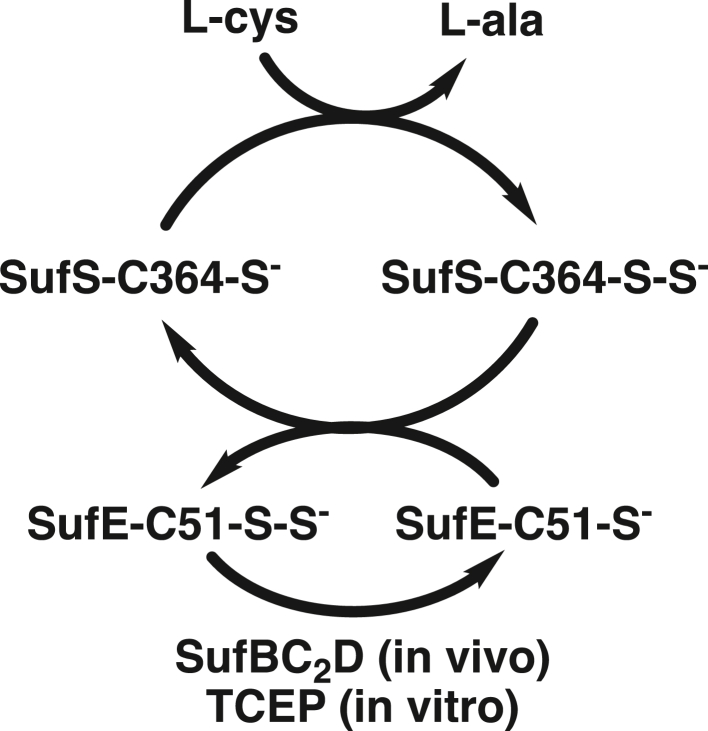
**Reaction cycle for the SufS cysteine desulfurase in the Suf pathway.**

IscS and SufS serve as examples of type I and type II cysteine desulfurases, respectively (4). Type I systems are defined by the long, flexible S-transfer loop containing the active-site cysteine and do not require a transpersulfurase partner. Type II enzymes are defined by a short, rigid active-site S-transfer loop and an insertion of amino acids that creates a β-hairpin motif. The β-hairpin reaches over and partially occludes the active site of the adjacent monomer (Fig. 2*A*). Type II enzymes, like SufS, are faced with a mechanistic challenge of stabilizing and protecting the active-site persulfide on a short S-transfer loop in a desulfurase step and making it available to a flexible S-transfer loop on SufE in a transpersulfurase step. Based on combined results from site-directed mutagenesis, backbone amide hydrogen–deuterium exchange, x-ray crystallography, and bioinformatics experiments, it has been proposed that a β-latch structural element regulates activity in type II cysteine desulfurases (8, 9, 10). The β-latch contains four key components: the α_6_ helix, a glycine-rich loop, a β-hairpin, and a *cis*-proline (Fig. 2*B*). The α_6_ helix contains three conserved residues. R92 and E96 (*E. coli* numbering) form cross-dimer electrostatic interactions with the α_6_ helix of the adjacent monomer, and N99 forms hydrogen bonds to the backbone amide and carbonyl of G251 in the glycine-rich loop (Fig. 2, *B* and *C*). The glycine-rich loop (G251/G252/G253) immediately proceeds the β-hairpin (256–266) with both components forming part of the active site of the adjacent monomer. The backbone amide of S254, located directly between these two components, can form interactions with the active-site persulfide on C364 (11). A conserved *cis*-proline (P271) concludes the motif and is thought to acts as a rigid joint around which the β-hairpin pivots.

**Figure 2:**
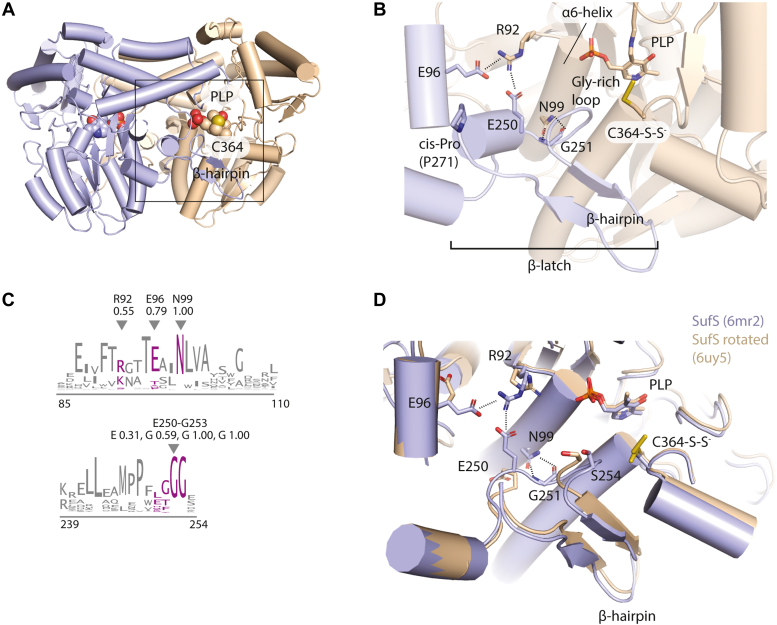
**Components of the β-latch regulatory motif in SufS.***A*, the SufS dimer (PDB code 6mr2) is displayed with blue and tan monomers. The 5′-pyridoxal phosphate cofactor (PLP) and residue C364 mark the active site. A rectangle indicates the area highlighted in *B*. *B*, components of the β-latch are labeled as well as key residues described in the text. *C*, a sequence logo indicates the conservation of key β-latch residues. Residues of interest are colored magenta and *E. coli* numbering is used in the sequence logo. The frequency for each residue of interest is denoted below the label. *D*, superposition of the WT SufS dimer in two different crystal forms. The blue structure is PDB code 6mr2, and the tan structure is PDB code 6uy5. Differences in the β-latch are highlighted.

The β-latch structural element is dynamic, and motion of the components results in a 3- to 4-Å rotation between the SufS monomers (captured in structures of both WT and variant enzymes) with several consequences (Fig. 2*D*) (8). The modulation results in disruption of the cross-dimer electrostatic interactions on the α_6_ helix and movement of the glycine-rich loop and β-hairpin away from the active site of the adjacent monomer, which may allow SufE closer access to the active site. There is also evidence that this modulation can shift the direction of the C364 persulfide from inward-facing to outward-facing (9). Importantly, backbone amide hydrogen-deuterium exchange mass spectrometry data show that formation of the C364 persulfide results in changes to deuterium uptake in both the α_6_ helix and glycine-rich loop, suggesting the β-latch is sensitive to the persulfide state of the enzyme (10). These structural changes are consistent with a role for the β-latch in the regulation of SufS. To investigate this hypothesis, site-directed mutagenesis was used to generate SufS variants at the conserved N99 residue. This residue physically links the α_6_ helix and G251 of the glycine-rich loop through hydrogen bonds (Fig. 2*B*). Structural results confirm that the substitutions cause changes in the glycine-rich loop and β-hairpin regions and significant effects on the cross-dimer interactions of the α_6_ helix. This perturbation results in enzyme variants that no longer require SufE for turnover and can instead react with exogenous reductant. Additional results suggest that disruption of the β-latch also precludes formation of a close approach SufS–SufE complex. These results are consistent with N99, and by extension the β-latch structural element, playing a key regulatory role in SufS function. As the β-hairpin motif is located in a similar position as the binding site for type I cysteine desulfurase regulatory proteins (*e.g.*, frataxin and CyaY), this site may be a conserved regulatory feature for cysteine desulfurases in general.

## Results

### Substitution of N99 results in perturbations to the β-latch structure and loss of persulfide protection

**Figure 3:**
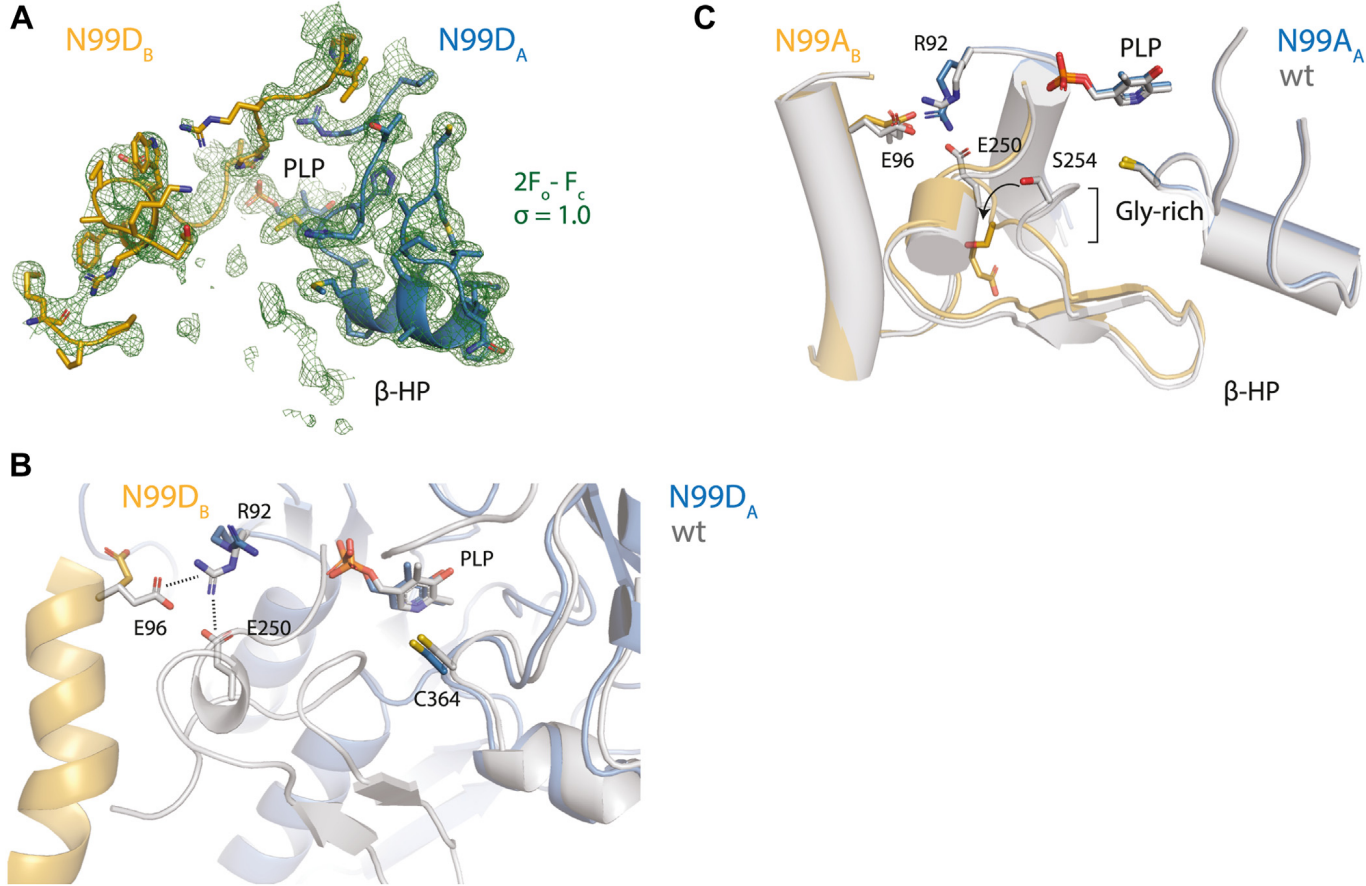
**Structural changes in N99 variants revealed by x-ray crystallography.***A*, a 2F_o_-F_c_ electron density map, contoured at 1 σ, of the SufS N99D variant. Disruption of the normal N99-Gly-rich loop hydrogen-bonding interactions has caused disordering of the β-hairpin (β-HP) indicated by a loss of electron density. *B*, a superposition of the structures of wt SufS (*gray*) and SufS N99D (*blue and tan*) shows electrostatic interactions at the dimer interface between R92 and E96 or E250, known to be functionally significant, are lost in SufS N99D. *C*, disruption of the N99-Gly-rich loop interactions in SufS N99A causes repositioning of the Gly-rich loop but not disordering of the β-HP. A superposition of wt SufS is shown with SufS N99A. An *arrow highlights* the rotation of the Gly-rich loop and two functionally important residues E250 and S254.

We hypothesized that substitutions of N99 that interfered with its hydrogen-bonding interactions with the glycine-rich loop would perturb the β-latch providing a tool to assay its contributions to enzyme function. We generated variants of SufS with either alanine or aspartate at position N99 and solved the crystal structures of the resulting proteins. Alteration of N99 results in significant, but localized, perturbations in components of the β-latch structural element. The most drastic effect is seen in the N99D SufS structure. In this structure, electron density is missing for residues 248 to 268, which includes the glycine-rich loop (G251-S254) and the β-hairpin (I256-T266) (Fig. 3*A*). Additional perturbations are seen for residues R92 and E96 on the α_6_ helix. In WT SufS, these two residues form a cross-dimer electrostatic interaction. The cross-dimer interaction between R92 and E96 is broken in N99D SufS, and the residue positions most closely resemble the open structure of WT SufS (Protein Data Bank [PDB] code 6uy5) with spontaneous shifts in the structure of the β-latch (Fig. 3*B*) (8). The structure of N99A SufS has continuous electron density for the β-latch; however, the glycine-rich loop and β-hairpin are shifted, exposing the SufS active site to solvent (Fig. 3*C*). In contrast to N99D, the N99A SufS structure does not show disruption of the cross-dimer interactions between R92 and E96. However, E250 is no longer able to participate in electrostatic interactions in the structure captured by crystallography.

**Figure 4:**
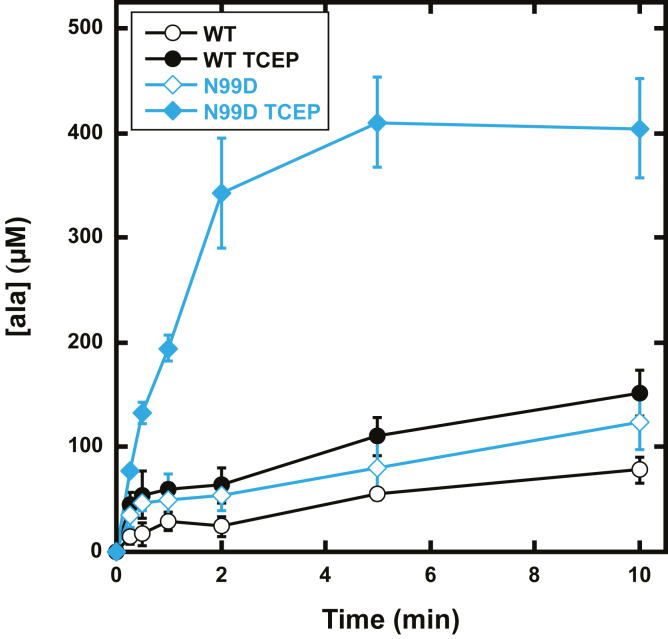
**Kinetics of alanine production by WT and N99D SufS in the absence of SufE.** Alanine production was measured by a plate reader–based alanine detection assay as described in Experimental procedures. Reaction conditions are 50 mM Mops (pH 8.0), 150 mM NaCl, 500 μM cysteine, and 30 μM SufS at 25 °C. Filled data points were collected in the presence of 2 mM TCEP. Open data points were collected in the absence of TCEP. *Solid lines* are a linear connection between each data point and do not represent a fit of the data. Error bars represent the standard deviation of at least three replicates.

With confirmation of structural perturbations to the β-latch caused by the N99 substitutions, we hypothesized that any protective function of the β-latch would also be disrupted. To test this hypothesis, 30 μM WT or N99D SufS were mixed with 500 μM cysteine in the absence of SufE and the presence or absence of 2 mM TCEP, and the formation of alanine was followed using a fluorescence-based plate-reader assay (Fig. 4) (12). In the presence of 500 μM cysteine and 2 mM TCEP, N99D SufS catalyzes significant production of alanine over a 10-min period (filled diamonds). Under identical conditions, WT SufS is also capable of producing alanine (filled circles), but less that N99D SufS. In the absence of TCEP (open symbols), both WT and N99D SufS exhibit near identical alanine production kinetics to the WT enzyme in the presence of TCEP. Results for this experiment demonstrate that N99D SufS no longer inhibits the active-site persulfide from reacting with TCEP, consistent with the model that a key biological role of the β-latch is to shield the active-site persulfide from off-pathway reactions.

### Steady-state kinetics indicate the β-latch affects SufS–SufE interactions

**Figure 5:**
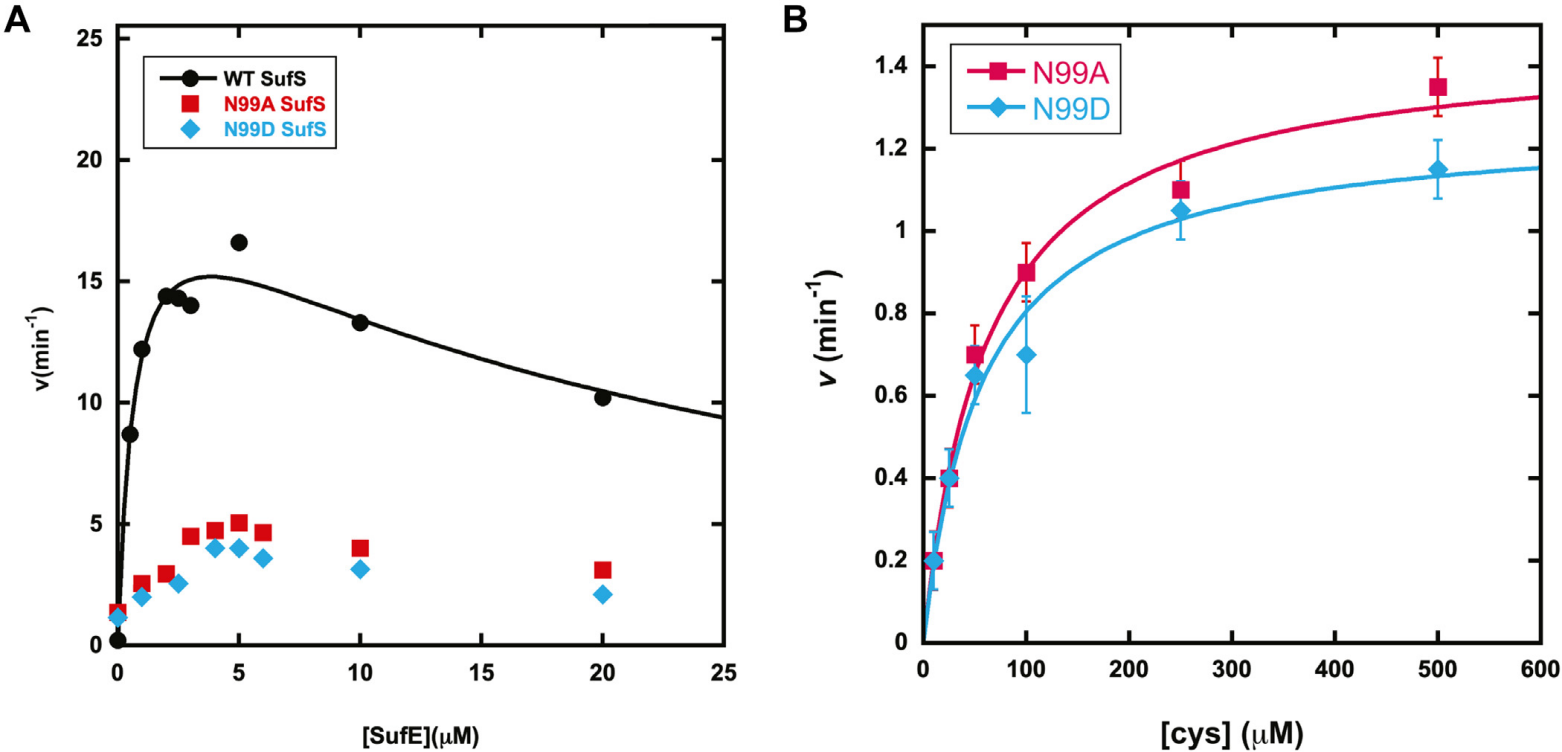
**Kinetics of alanine production by WT and variant SufS.** Alanine production was measured by an HPLC-based assay as described in Experimental procedures. Data are shown for rates of alanine formation for WT (*black circles*), N99A (*red squares*), and N99D (*blue diamonds*) SufS. Reaction conditions are 50 mM Mops (pH 8.0), 150 mM NaCl, 2 mM TCEP, and 0.1 to 1.0 μM SufS. When SufE is present, SufS concentrations were at least 5-fold lower than the SufE concentration in each assay. *A*, initial velocities determined by varying SufE concentrations in the presence of 500 μM cysteine. The solid black line is from a fit of the data to Equation 2. *B*, initial velocities determined by varying cysteine concentrations in the absence of SufE. *Solid lines* are produced by a fit to Equation 1. Error bars represent the standard deviation of at least two replicates and are smaller than the data point if not shown.

Steady-state kinetics were followed by an HPLC-based fluorescence alanine detection assay to better quantitate the effect of β-latch perturbations on the catalytic function of SufS. The reactions reported in this section contain 2 mM TCEP, which is required for the regeneration of SufE in the WT system (Fig. 1) (6). In our hands, the WT enzyme displays kinetic parameters similar to those previously reported (6, 10) with an apparent *k*_cat_ value of 22 ± 2 min^-1^ and apparent *K*_m_ values of 0.8 ± 0.1 μM and 63 ± 4 μM for SufE and cysteine, respectively (Table S1). At SufE concentrations higher than 5 μM the activity of WT SufS decreased, and the overall kinetics are best fit by an equation including substrate inhibition (Fig. 5*A*). Similar SufE substrate inhibition kinetics for SufS have been previously reported, albeit at lower cysteine concentrations (7). The kinetics of SufE dependence for N99A and N99D SufS is complicated with both enzymes displaying a linear dependence on SufE concentrations up to 5 μM with inhibition occurring above 5 μM SufE (Fig. 5*A*). Owing to the linear dependence of the velocity at low [SufE], the Michaelis–Menten plots for the N99 variants are not well fit by an equation including substrate inhibition. The activity of the N99 variants in the absence of SufE is consistent with the TCEP-dependent activity described in the preceding section. The N99 substitutions do not appear to affect interactions with cysteine as N99A and N99D SufS exhibit Michaelis–Menten kinetics in response to varying cysteine concentrations in the absence of SufE with apparent *K*_M_ values of 56 ± 10 μM and 62 ± 8 μM, respectively (Fig. 5*B*). These values are similar to those determined for WT SufS in the presence of SufE. The steady-state activity of N99D SufS was also measured under various concentrations of TCEP to address the mechanism of interaction (Fig. S1). While increasing TCEP concentrations caused an increase in the rate of alanine formation, the data do not allow for discrimination between a second-order, collisional mechanism (linear dependence) and a mechanism that involves a saturable binding step prior to reduction (hyperbolic dependence).

### A perturbed β-latch does not affect formation of initial cysteine-PLP intermediates

**Figure 6:**
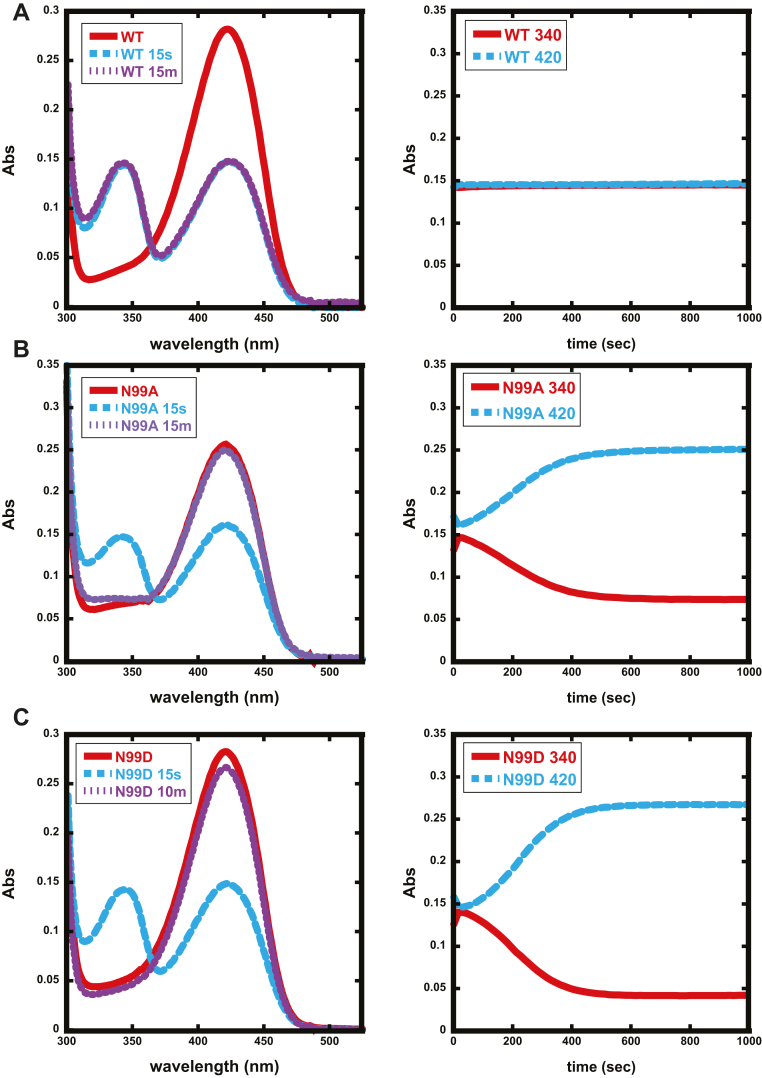
**The reaction of WT SufS and N99 variants with cysteine in the absence of SufE monitored by UV-visible absorption.***A*, Panels on the left of the figure show UV-visible spectra of WT or *B*, *C*, variant SufS prior to addition of cysteine (*red*) and at 15 s (*blue*) and 15 min (*purple*) after cysteine addition. Panels on the right show extracted time-dependent single-wavelength traces for the peaks corresponding to 340 (*red*) and 420 nm (*blue*).

To determine if the β-latch plays a role in the reaction of cysteine with PLP, the reaction catalyzed by N99 variants of SufS was followed by UV-visible spectroscopy. Cysteine was mixed with 30 μM SufS in the presence of 2 mM TCEP to monitor formation of cysteine-PLP intermediates along the reaction pathway in a photodiode array spectrophotometer. The as-purified enzymes exhibit an initial peak at ∼420 nm, representing the internal aldimine species. As previously reported, addition of cysteine to WT SufS results in the rapid (<5 s) reduction of the ∼420 nm peak by 50% (13). Concomitant with loss of the ∼420 nm peak, a new peak at 343 nm is formed (Fig. 6*A*). The enzyme requires 2 to 3 h for the 343 nm peak to disappear and the full 420 nm peak to be restored. Previously, the two prominent SufS absorbance peaks after addition of cysteine were hypothesized to result from formation of cys-ketoenamine (∼420 nm) and cys-enolimine (∼343 nm) resonance structures of a stalled external cys-aldimine SufS species (13). With the demonstration that WT SufS completes multiple turnovers in the absence of SufE (albeit very slowly), these species are better characterized as the predominant intermediates under steady-state conditions (*i.e.*, intermediates preceding slow steps in the reaction) in the absence of SufE. When cysteine is added to N99D SufS, a rapid decrease of ∼50% is seen at ∼420 nm with a corresponding increase at ∼340 nm, similar to the results with the WT enzyme (Fig. 6*B*). However, the species do not persist, and the initial 420 nm absorbance state is regained after only 10 to 15 min rather than the 2-h time frame for the wildtype enzyme. N99A SufS exhibits results similar to N99D SufS (Fig. 6*C*). The rapid shift back to the 420-nm species is consistent with the kinetic data in Figure 4 showing N99D rapidly converts cysteine to alanine in the presence of TCEP. When combined with the steady-state kinetics results, it appears that substitution of N99 has not substantially affected the interactions of SufS with cysteine or the reactivity of the PLP cofactor. Instead, the kinetic defects resulting from a perturbed β-latch are localized to interactions with SufE.

### β-Latch perturbations affect the interactions of SufS with SufE

**Figure 7:**
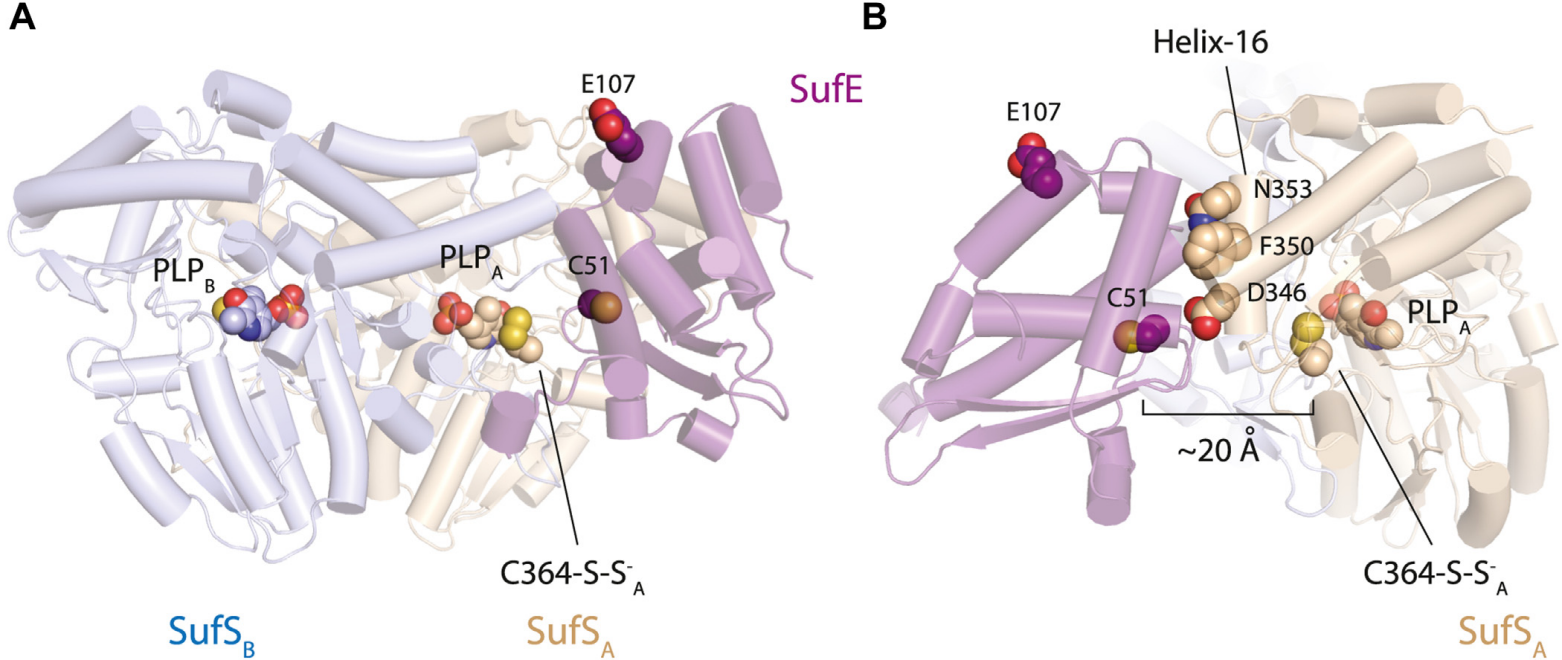
**A computational model of the SufS–SufE initial-encounter complex.***A*, Rosetta was used to dock the crystal structures of SufE, given by PDB code 1mzg, and SufS given by PDB code 6mr2. SufE is shown in purple with SufS in blue and tan. PLP and C364-persulfulde are shown as spheres to mark the active site, and SufE residues of interest, C51 and E107, are also shown as spheres. *B*, a close-up view of the docked SufS–SufE interaction reveals that the α_16_ helix of SufS forms extensive noncovalent interactions with SufE. The computational model of the SufS–SufE complex is an initial-encounter model because C51, the active-site Cys of SufE, is too far (∼20 Å) from C364 of SufS for persulfide transfer.

The altered steady-state kinetics measured when varying SufE suggested that the β-latch might play a role in SufS binding to SufE. As a first step in answering this question, we constructed a RosettaDock model of SufS bound to SufE since no crystal structure of the complex exists. The best scoring model from this computation had an orientation of SufS and SufE similar to the crystal structure of the cysteine desulfurase CsdA bound to the transpersulfurase CsdE reported in PDB code 4lw4 (Figs. 7*A* and S2) (14). In both cases the transpersulfurase (SufE or CsdE) interacts extensively with an α-helix in the cysteine desulfurase (termed α_16_) that sits above the active site and points toward solvent (Fig. 7*B*). The active-site cysteines in the cysteine desulfurase and transpersulfurase point toward each other but are too far apart in space for sulfur transfer consistent with the identification of additional, possibly transient, conformational changes in SufS and SufE necessary for the chemistry (15, 16). The computational model likely represents an on-pathway, initial encounter complex that is populated just before the conformational change that facilitates chemistry. However, the computational model is useful because it suggests specific residues in the α_16_ helix of SufS (N353, F350, and D346) whose interactions with SufE drive binding.

**Figure 8:**
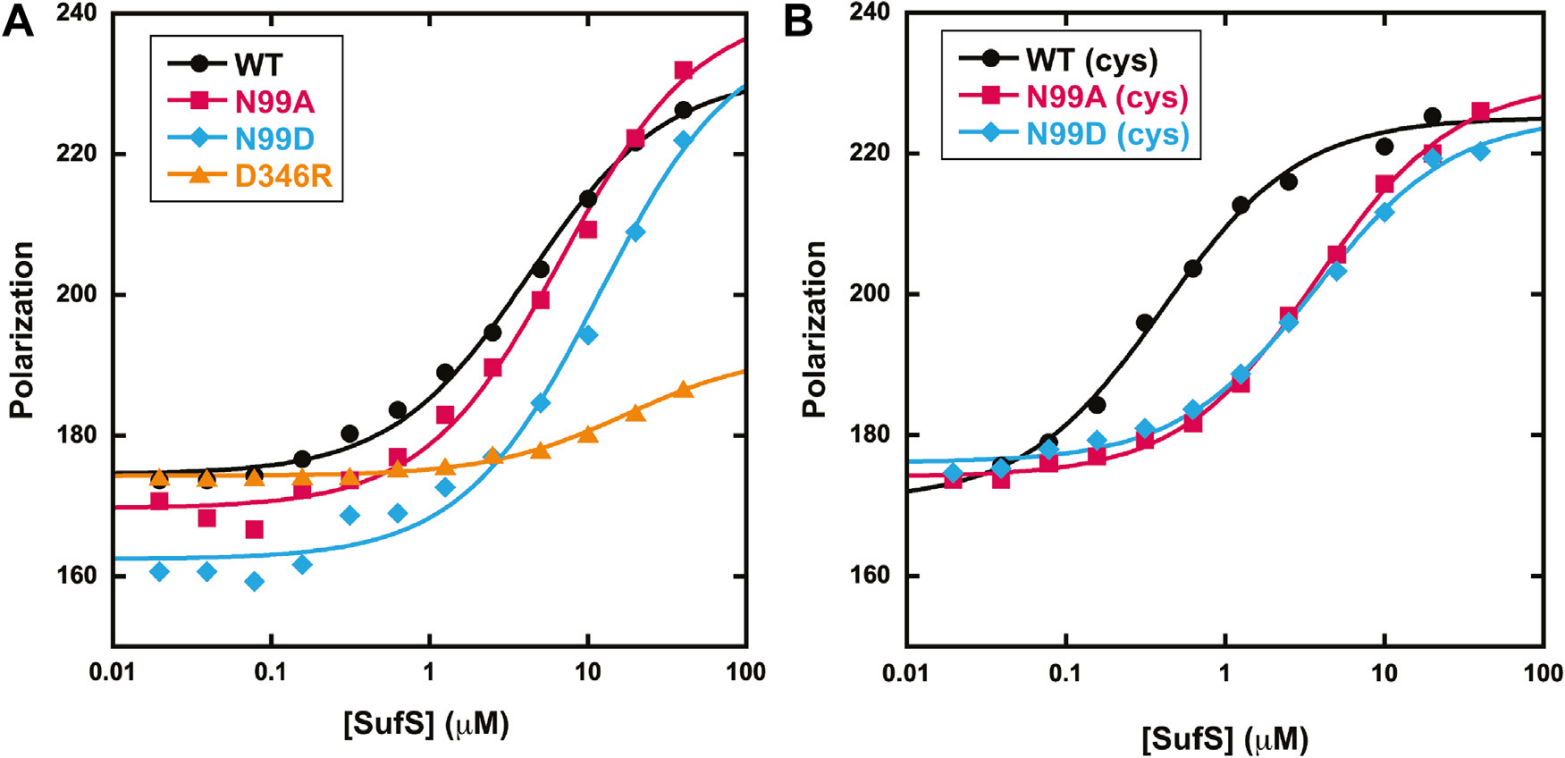
**SufS–SufE binding measured by fluorescence polarization.***A*, representative data for changes in fluorescence polarization due to titration of labeled C51A/E107C SufE by WT (*black circles*), N99A (*red squares*), N99D (*blue diamonds*), or D346R (o*range triangles*) SufS. *B*, representative data for changes in fluorescence polarization due to titration of labeled C51A/E107C SufE by WT (*black circles*), N99A (*red squares*), or N99D (*blue diamonds*) SufS in the presence of 500 μM cysteine. *Solid lines* in both panels are from a fit of the data to Equation 3.

The model supports the rational design of SufS mutants with disrupted binding to SufE, a useful tool in developing and validating SufS–SufE binding assays. To this end, a SufS/SufE binding assay based on fluorescence polarization was developed such that a fluorescently labeled SufE should show an increase in fluorescence polarization upon formation of the larger SufS–SufE complex. Using the Rosetta SufS–SufE model as a guide, E107 was selected as a position in SufE that could be covalently labeled with BODIPY-maleimide dye without disrupting SufE function. Thus, E107 and the persulfide accepting C51 were substituted to create a C51A/E107C variant of SufE to serve as a reagent in the fluorescence polarization binding assay. A titration of WT SufS against labeled C51A/E107C SufE results in an increase in fluorescence polarization (Fig. 8*A* and Table 1). The data can be fit to Equation 3, which describes a single-affinity binding site to determine a *K*_D_ value of 5.2 ± 1.2 μM, consistent with binding affinities measured by other methods (16). D346R SufS, a substitution predicted to abrogate SufS–SufE binding based on a Rosetta-derived model of the protein complex, was developed to test the assay (Fig. 8*A*, yellow triangles). Consistent with the prediction above, titration of the D346R SufS shows limited increases in fluorescence polarization under conditions where the wildtype proteins show substantial interactions and suggests a *K*_D_ value greater than 40 μM, the highest concentration of SufS used in the assay.

**Table 1:** Dissociation constants for SufS–SufE interactions^a^

SufS	*K*_D_ in absence of cysteine (μM)	*K*_D_ in presence of cysteine (μM)
WT	5.2 ± 1.5^b^	0.6 ± 0.2
N99A	6.2 ± 1.8	3.1 ± 0.6
N99D	9.9 ± 3.0	3.3 ± 0.7

a Determined as described in Experimental Procedures.

b Mean value determined from independent experiments ± propagated standard error.

Figure 8*A* also shows the fluorescence polarization results for a titration of N99A and N99D SufS against labeled SufE. The N99 variant enzymes are able to interact with SufE with low micromolar K_D_ values similar to the value determined for the WT SufS–SufE interaction (Table 1). This result suggests that the N99 substitutions have not disrupted the initial-encounter complex between the two proteins. However, previous studies have shown that a C51-alkylated SufE binds to SufS with *K*_D_ value of 0.5 μM (10). This 10-fold increase in affinity is hypothesized to occur because the alkylated SufE represents a better mimic of the functional interaction that results in persulfide transfer. In an attempt to mimic this functional interaction, 500 μM cysteine was added to the titration conditions. WT SufS will process cysteine slowly and will build up the steady-state intermediates as shown in Figure 6*A*, which could be trapped by the inactive BODPIY-C51A/E107C SufE. In the absence of TCEP, the N99 variants should behave in a similar manner. In the presence of 500 μM cysteine the WT SufS–SufE interaction is dramatically improved to a value of 0.6 ± 0.2 μM, consistent with the value determined with C51-alkylated SufE (Fig. 8*B* and Table 1) (10). This suggests that SufE is able to probe the higher-affinity “close approach” functional interaction between SufS and SufE when cysteine is present. Under identical conditions, the two N99 SufS variants show only modest improvement in their *K*_D_ values for SufE, and the values remain similar to those determined in the absence of cysteine. These results suggest that, when the β-latch of SufS is perturbed, SufE can still bind in an initial-encounter complex but cannot form the close approach conformation, the structure of the complex required to promote efficient persulfide transfer. This finding is consistent with the altered steady-state kinetics parameters described above.

## Discussion

Cysteine desulfurases are ubiquitous PLP-dependent enzymes responsible for the mobilization of sulfur from cysteine for use in the biosynthesis of multiple biological compounds (4). The type II cysteine desulfurases are defined by a short, rigid S-transfer loop and a β-latch structural element that forms part of the active site on the adjacent monomer. Owing to the short S-transfer loop, type II desulfurases require accessory transpersulfurase proteins to transfer the covalent persulfide to downstream protein partners. Although the β-latch appears to be ubiquitous in type II enzymes, there is not a clear understanding of the functional role of this evolutionarily conserved feature. To answer this question, site-directed mutagenesis was used to generate variants of SufS from *E. coli* targeted to a conserved asparagine residue (N99), which forms a key interaction in the β-latch.

### The β-latch provides a protective mechanism for the *E. coli* SufS persulfide

As described above, a key feature of *E. coli* SufS (and the *E. coli* Suf pathway in general) is its ability to protect reactive persulfide intermediates under harsh cellular conditions (6, 7). The most striking result from this work is the loss of that protection in the N99 SufS variants as evidenced by the robust alanine production using TCEP as a persulfide reductant. These kinetic results are readily explained when the structures of the N99 variants are examined. Disruption of the N99 interactions with the β-latch results in changes to all facets of the motif. One can hypothesize that the changes most relevant to the protective function of the β-latch are the disruption of the interaction between the N99 position and the backbone of the glycine-rich loop, which makes up a wall of the active-site opening. In the N99D SufS structure, the loss of density for the glycine-rich loop and the β-hairpin suggests increased solvent access to the active-site persulfide (Fig. 3*A*). In the N99A SufS structure density for the glycine-rich loop and the β-hairpin is seen, suggesting they are less dynamic and are still capable of physically blocking the active site from solvent. However, the glycine-rich loop is in an altered conformation compared with the WT SufS structure (Fig. 3*B*). This altered conformation shifts the position of the S254 amide away from the active site by over 8 Å. The backbone amide of S254 has been shown to interact with the terminal sulfur of the C364 persulfide in the outward-facing position (11). Loss of this interaction could affect the stability of the persulfide intermediate to nucleophilic attack.

### The β-latch is also responsible for productive SufS–SufE interactions in *E. coli*

While the protective nature of the *E. coli* SufS–SufE system is critical for its biological function, the β-latch is conserved in all type II cysteine desulfurases (8). It is not clear if the protective mechanism is exhibited in systems where it is not specifically required (*i.e.*, systems that do not operate under conditions of oxidative stress). This raises the possibility of a more general function of the β-latch in type II cysteine desulfurases. As described above, type II cysteine desulfurases require an accessory transpersulfurase protein to transfer the covalent persulfide intermediate. The best models for the SufS–SufE interaction come from costructures of the homologues CsdA–CsdE in *E. coli* (17, 18) and the SufS–SufU costructure from *Bacillus subtilis* (19). Both structures show key interactions between the desulfurase and the transpersulfurase occurring in the α_16_ helix region of the desulfurase (residues 344–354 in *E. coli* SufS). In the current costructures the distance between the persulfide donors and acceptors is too far to support efficient persulfide transfer. This suggests that additional conformational changes are required to create a “functional” interaction.

**Figure 9:**
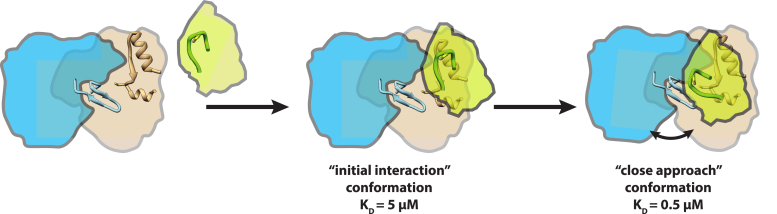
**Model for SufS–SufE interactions.** Initial SufS (*blue and tan*) and SufE (*green*) interactions involve formation of an “initial interaction” complex centered on binding of SufE to the α_16_ helix on SufS. Upon formation of the SufS persulfide and rearrangement of the beta-latch, a “close approach” conformation is formed with new interactions between SufE and the beta-hairpin motif.

Based on homologous structures and the Rosetta model, the β-hairpin motif lies ∼10 Å across from a face of the SufE protein, including the region directly upstream of the persulfide accepting cysteine. It is possible that rotation around the SufS α_16_ helix–SufE interaction axis could close the gap between the two proteins to form a fully functional interaction capable of protected and efficient persulfide transfer. The data presented here and in previous reports support this two-step interaction model (Fig. 9). The D346R SufS variant exhibits severely diminished interactions with SufE under standard assay conditions, consistent with the α_16_ helix region mediating initial SufS–SufE interactions. In the absence of cysteine, WT and the N99 SufS variants bind to SufE with low micromolar *K*_D_ values representative of the initial interaction and likely represented by homologous desulfurase–transpersulfurase costructures. The addition of cysteine to WT SufS allows for formation of the functional SufS–SufE complex and a 10-fold decrease in the *K*_D_ value. However, with the structural disruptions in the β-hairpin region caused by the N99 substitutions, the SufS variants are not able to form this functional complex and exhibit minor improvements in their *K*_D_ values.

### Comparison of the type II β-latch-mediated SufS–SufE interaction with type I systems identifies a regulatory hotspot

**Figure 10:**
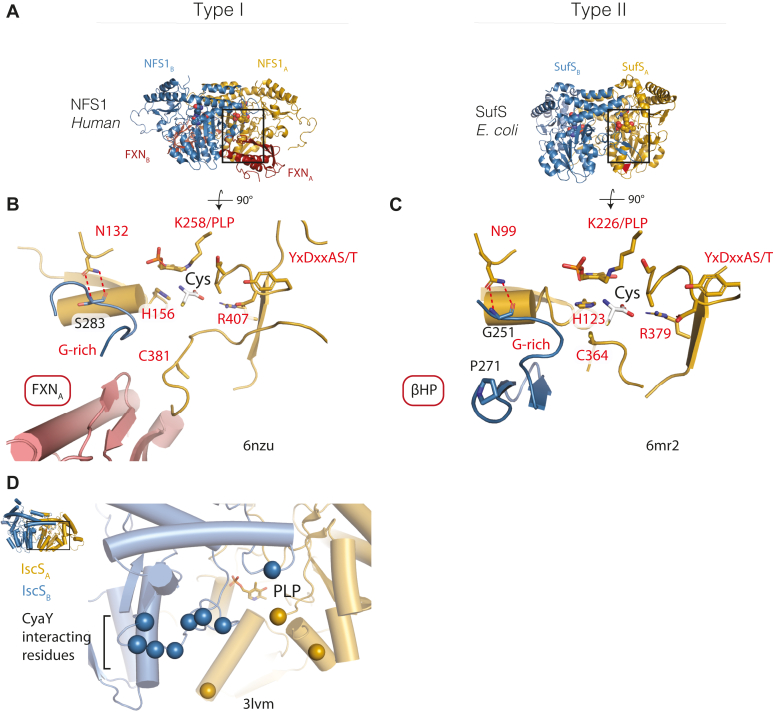
**Common features in the active sites of type I and type II cysteine desulfurases.***A*, structural comparison of human NFS1, a type I cysteine desulfurase, bound to the accessory protein Frataxin (FXN) and an overview of the structure of *E. coli* SufS, a type II cysteine desulfurase. *B*, detailed image of type I NFS1 active site (boxed region in *A*). The human NFS1 (PDB code 6nzu, *B*) contains a hydrogen-bonding interaction between N132 and a Gly-rich region adjacent to its active site marked by PLP. Residues that reside in positions known to be important for the chemistry of prokaryotic cysteine desulfurases are shown: H156 (acid–base catalysis), K258 (PLP binding), R407 (Cys binding), and C381 (persulfide carrier). Additional conservation is seen in the right wall of the active site (YxDxxAS/T motif). The frataxin protein (FXN) is shown in red. The Cys substrate (*white*) is modeled into the active site for visual reference. *C*, detailed image of type II SufS active site (boxed region in *A*). The active site of SufS, given by PDB code 6mr2, has many features in common with the human NFS1 active site, highlighted by shared red labels, with the exception of the βHP. The βHP can be seen to occupy a similar space to the accessory protein frataxin in the NFS1 structure in *B*. The Cys substrate (*white*) is modeled into the active site for visual reference. *D*, a close-up view of the active site of the type I cysteine desulfurase, IscS (PDB code 3lvm), is shown with spheres marking residues demonstrated to mediate interactions with the Frataxin homology, CyaY.

As described above, cysteine desulfurases can be described as type I and type II systems (Fig. 10*A*). The two types contain similar active-site architecture (Fig. 10, *B* and *C*) but are differentiated by a relative sequence insertion resulting in a flexible S-transfer loop for the persulfide-containing cysteine in type I enzymes compared with a buried, inflexible S-transfer loop in type II enzymes. The flexible S-transfer loop in the type I enzymes allows for direct transfer of the persulfide to acceptors, while the type II systems require an accessory transpersulfurase with a flexible S-transfer loop containing the persulfide acceptor. Both systems share the common challenge of creating a complex capable of transferring the persulfide intermediate between the donor and acceptor cysteines with one cysteine residing on a flexible loop. In the type II systems, we propose that the β-latch is responsible for mediating interactions by guiding the flexible persulfide acceptor loop on SufE toward the donor cysteine on SufS to promote a functional interaction (Fig. 10*C*). In type I cysteine desulfurases, the challenge is guiding the flexible S-transfer donor loop to specific S-transfer acceptors. In NFS1, the type I mitochondrial cysteine desulfurase, this challenge is overcome by the accessory protein frataxin. Frataxin is required for optimal function of NFS1 and has been shown to kinetically promote persulfide transfer (20) and structurally guide the flexible S-transfer loop for functional interactions with the acceptor protein ISCU (Fig. 10*B*) (21). The type I bacterial cysteine desulfurase, IscS, is inhibited by interactions with CyaY, a frataxin homologue, and requires IscX to disrupt the IscS–CyaY interaction (22). A previously reported mutagenesis screen of IscS identified several residues essential for binding of CyaY (23). A comparison of IscS and SufS shows these residues occupy the same space as the β-latch (Fig. 10*D*). Surprisingly, the mode of activation or inhibition by the accessory protein is encoded by the desulfurase structure as frataxin and CyaY both activate NFS1 and both inhibit IscS (24).

These commonalities suggest that the type I and type II cysteine desulfurases utilize a similar structural feature adjacent to the active site to coordinate persulfide transfer between donor and acceptor S-transfer loops. Type I enzymes, which are capable of interacting with multiple acceptor proteins, can be regulated by forming a ternary complex with various acceptor and accessory proteins to promote specific conformations of the S-transfer donor loop. Type II cysteine desulfurases have dedicated persulfide acceptors and utilize the native β-hairpin, rather than exogenous accessory proteins, to promote S-transfer donor–acceptor loop interactions.

## Conclusions

The presence of a conserved β-hairpin structure is a defining characteristic of type II cysteine desulfursases. Recent kinetic, structural, and bioinformatic results proposed the β-hairpin is part of a larger, connected set of features dubbed the β-latch structural element. Here we have shown that the conserved N99 residue acts as a linchpin in the β-latch. Substitution of this residue in SufS from *E. coli* resulted in a kinetically impaired enzyme. More in-depth characterization showed the N99 enzyme variants lost the native protection of the covalent persulfide intermediate to external reductants and could no longer form a high-affinity, functional SufS–SufE complex. Structures of the two SufS variants showed these functional defects could be explained by perturbations to the β-latch. Comparison of the location of the β-latch with regulatory accessory proteins of type I cysteine desulfurases suggests a conserved architectural feature in cysteine desulfurases plays an important regulatory role in selecting specific conformations of the S-transfer donor and acceptor loops in all cysteine desulfurases.

## Experimental procedures

### Generation of the SufS and SufE variants

Mutagenesis of the SufS (Uniprot ID:P77444) or SufE (Uniprot ID:P76194) gene in vector plasmid pET-21(a) was accomplished using Quikchange Lightening Mutagenesis Kit (Agilent) for SufS and inverse PCR for SufE. Forward and reverse primers with desired mutations were introduced to obtain the N99A, N99D, and D346R SufS variants as well as the C51A/E107C SufE variant (Table S2). The resulting plasmids were transformed into XL-10 Gold *E. coli* cells, and the purified plasmid was sent for DNA sequencing (Eurofin Genomics) to ensure the desired mutation.

### Protein expression and purification

SufS and SufE enzymes were expressed and purified as discussed (10). For recombinant protein expression, sequenced pET21 plasmids carrying the desired gene sequence were transformed into electrocompetent BL21(DE3)*Δsuf E. coli* cells. Cells were grown overnight in Luria Broth with 100 μg/ml ampicillin and 50 μg/ml kanamycin at 37 °C. To initiate 1-L cultures, the overnight growth was diluted to 100-fold in LB and allowed to incubate at 37 °C until the cells reached an *A*_600_ of 0.4 to 0.6. Protein expression was then induced using 500 μM isopropyl-β-D-thiogalactoside. Cells were grown for 3 h post induction, harvested via centrifugation, and cell pellets were stored at −80 °C. To begin purification, cell pellets were thawed and resuspended in 25 mM Tris pH 7.5, 100 mM NaCl, 5 mM dithiothreitol (DTT), 1 mM phenylmethylsufonyl fluoride, 1 mM DNase, and 1 mM MgCl_2_. The resuspended cells were lysed via sonication, and cellular debris was pelleted via centrifugation. The clarified lysate containing SufS was subjected to purification using three different columns: Q-XL, Phenyl HP, and Superdex 200 (Cytivia). The Q-XL column used a linear gradient from 25 mM Tris-HCl, pH 7.5, 10 mM β-mercaptoethanol (BME) to 25 mM Tris-HCl, pH 7.5, 10 mM BME, 1 M NaCl. The Phenyl HP column used a linear gradient from 25 mM Tris-HCl, pH 7.5, 100 mM NaCl, 1 M (NH_4_)_2_SO_4_, 10 mM BME to 25 mM Tris-HCl, pH 7.5, 10mM BME. The Superdex 200 column was run with 25 mM 3-(N-morpholino)propanesulfonic acid (MOPS), pH 7.5, 150 mM NaCl, 10 mM BME. Lysate containing SufE was purified using Q-XL and Superdex 200 columns. Fractions from all the columns containing the protein of interest were identified using SDS-PAGE. Selected fractions were pooled, concentrated, and stored in gel filtration buffer supplemented with 10% glycerol at −80 °C for further use. SDS-PAGE was used to assess the purity of the final sample (Fig. S3). SufS concentration was determined based on quantification of PLP at 388 nm in 0.1 M NaOH (ε_388_ = 6600 M^−1^ cm^−1^) (25). SufE concentration was determined with a calculated extinction coefficient of ε_280_ = 20,970 M^−1^ cm^−1^.

### Alanine-detection assay

Cysteine desulfurase activity was measured by monitoring L-alanine production in a naphthalene 2,3-dicarboxaldehyde (NDA) labeled reaction (12). The assay mixture contained the following components: 0.25 to 1 μM SufS (based on PLP absorbance), 100 mM MOPS, pH 8.0, 150 mM NaCl, 2 mM TCEP, and varying amounts of SufE and cysteine. For the HLPC-based assay, a 700-μl reaction was initiated by addition of SufS and, at various time points, 100-μl aliquots were quenched using 20 μl of 10% trichloroacetic acid. This was followed by adding 1 ml of NDA labeling mix (100 mM borate, pH 9.0, 2 mM KCN, and 0.2 mM NDA) and incubating the reaction mixtures in the dark for 60 min. After the incubation, 200 μl of the reaction mixtures was transferred to vials in the HPLC autosampler. The autosampler was set to inject 10 μl of each sample onto a Zorbax C18 column (Agilent) at a flow rate of 0.4 ml/min with an isocratic gradient of 50% 10 mM ammonium acetate with 50% methanol (pH 6.0) for 11 min. The fluorescent adduct was measured with a Shimadzu HPLC coupled with a florescence detector set to 390 nm excitation and 440 nm emission. Alanine standards were also prepared under the same conditions to construct a standard curve. The alanine-NDA peak was integrated, and the peak area was converted into nanomoles of alanine using the standard curve. For the plate-reader assay, reactions were run using similar conditions to the HPLC-based assays. Reaction aliquots of 50 μl were quenched with 5 μl of 10% trichloroacetic acid and mixed with 200 μl of the NDA labeling mix. After a 20-min incubation with the NDA labeling mix in the dark, the fluorescence (390 nm excitation, 440 nm emission) for each time point was measured using a Biotek Synergy2 multiwell plate reader.

### Fluorescence anisotropy SufE binding assay

The pET21 plasmid carrying the C51A/E107C SufE gene was transformed into BL21(DE3) *E. coli* cells and expressed and purified as described for SufE above with a change in purification buffer pH from 7.5 to 8.0. Following purification and concentration, the protein was dialyzed into 50 mM MOPS (pH 8) and 150 mM NaCl to remove DTT. Fluorescently labeled samples of C51A/E107C SufE were created by incubating 50 μM protein with 500 μM BODIPY FL maleimide (Invitrogen) for 4 h at 25 °C. After the incubation period, unreacted dye was removed using a PD-10 column. Labeling efficiency was determined by comparing the ratio of label concentration (ε_505_ = 80,000 M^−1^ cm^−1^) with protein concentration (ε_280_ = 20,970 M^−1^ cm^−1^) and was determined to be between 60 and 80% using this method. SufS–SufE binding titrations were carried out in black 96-well plates in 50 mM MOPS (pH 8), 150 mM NaCl, and 0.1 mg/ml bovine serum albumin. SufS (0.2–40 μM) was mixed with 100 nM labeled C51A/E107C SufE and allowed to equilibrate at room temperature for 30 min. After equilibration, the fluorescence polarization (480 nm excitation, 520 nm emission) was measured using a BioTek Synergy2 multiwell plate reader.

### Crystallography methods

Crystallization and structure solution of SufS site-directed mutants was performed essentially as reported (9). Briefly, 8 to 9 mg/ml protein was mixed 2:1 (vol/vol) with crystallization solution, 4 to 4.5 M NaCl, and 100 mM MES pH 6.5, and subjected to sitting drop vapor diffusion at 20 °C. Crystals appeared in 1 to 7 days. Crystals were cryoprotected by soaking for several minutes in a solution consisting of mother liquor with 50% glycerol. Crystals were plunge-frozen in liquid nitrogen and x-ray data collection proceeded at 100 K with 1.54-Å x-rays generated by a Rigaku Synergy DW equipped with a HyPix6000-HE detector. Data reduction was performed with XDS (SufS N99D) or with Rigaku CrysAlis Pro software (SufS N99A). The structure given by PDB code 6mr2 (with heteroatoms removed) provided phases for structure solution. Model building and refinement were performed iteratively using Coot and Phenix. X-ray data statistics and model quality data are reported in Table S3. Final coordinates were submitted to the PDB as PDB codes 7ruj (N99A SufS) and 7rw3 (N99D SufS).

### Rosetta model construction

A computational model of SufE bound to SufS was constructed using RosettaDock in the local docking mode in Rosetta version 3.11. The SufS coordinates given by PDB code 6mr2 and the SufE coordinates given by PDB code 1mzg were prepared in the following manner. The SufS homodimer sits on a crystallographic symmetry axis in the crystal form reported in 6mr2. Therefore, the symmetry mate of SufS was generated in Pymol from the 6mr2 coordinates to produce the SufS homodimer with chains labeled A and B. Both the SufS coordinates and the SufE coordinates were cleaned (stripped of heteroatoms), renumbered, and repacked in Rosetta. SufE was positioned approximately 10 Å from SufS with its active site, marked by residue C51, facing toward the SufS active site in monomer A, marked by the SufS residue C364. The known, enzymatic mechanism of persulfide transfer from SufS to SufE dictates the two active-site cysteines must be physically adjacent to each other, which informed the starting position for SufE in the calculation. In addition, homologous structures such as SufS–SufU (PDB code 5xt6) and CsdA–CsdE (PDB code 4lw4) confirm the active sites of SufS and SufE must be physically adjacent to each other for persulfide transfer to occur. Rosetta was launched with command:

rosetta/3.11/main/source/bin/docking_protocol.default.linuxgccrelease

@flag_local_docking

using the flag_local_docking file:

- in:file:s 1mzg_6mr2_repack.pdb
- unboundrot 1mzg_6mr2_repack.pdb
- nstruct 1000
- partners AB_C
- dock_pert 3 8
- ex1
- ex2aro
- out:path:all Output_files
- out:suffix _local_dock

A plot of the interface score (I_sc) versus the interface rms difference (I_rms) among the decoys was generated, which identified Model 0014 as the lowest energy structure, existing at the nadir of a cluster of related structures in the plot (Fig. S3). Model 0014 is publicly available at https://dunklelab.ua.edu/structures.

### Data analysis

Nonlinear regression fits of the data to various equations were performed in Kaleidagraph 5.0 (Synergy Software). Initial velocities at various substrate concentrations were determined using a minimum of three time points. Initial velocity data were then fit to the Michaelis–Menten equation (Equation 1) or an equation describing substrate inhibition (Equation 2) to determine the kinetic parameters for each enzyme. Here, *v* is the initial velocity, *E*_t_ is the total enzyme concentration, *k*_cat_ is the maximal turnover number, *S* is the substrate concentration, *K*_M_ is the Michaelis constant, and *K*_i_ is the inhibition constant.

(1) $$\frac{v}{E_{t}}=\frac{k_{cat}\;\left[S\right]}{K_{M}+\left[S\right]}$$

(2) $$\frac{v}{E_{t}}=\frac{k_{cat}\;\left[S\right]}{K_{M}+\left[S\right]\left(1+\frac{\left[S\right]}{K_{i}}\right)}$$

Fluorescence polarization data used to measure protein–protein interactions were fit to Equation 3 where *A*_o_ is the polarization in the absence of the ligand, *ΔA* is the total change in polarization, and *K*_D_ is the dissociation constant.

(3) $$Fl\;polarization=A_{0}+\frac{\Delta A\times \left[SufS\right]}{K_{D}+\left[SufS\right]}$$

## Data availability

All kinetic and biophysical data are contained within the article and supporting information. Structural data have been deposited in the PDB under accession codes 7ruj for N99A SufS and 7rw3 for N99D SufS.

## Supporting information

This article contains supporting information.

## Conflict of interest

The authors declare that they have no conflicts of interest with the contents of this article.
