# Supplementary material for: The β-latch structural element of the SufS cysteine desulfurase mediates active site accessibility and SufE transpersulfurase positioning
Source: J Biol Chem. 2023 Feb 1;299(3):102966. doi: 10.1016/j.jbc.2023.102966 (PMC10011822; doi:10.1016/j.jbc.2023.102966)
Supplement: Supporting information [file mmc1.docx]

The β-latch structural element of the Suf pathway cysteine desulfurase, SufS, mediates active site accessibility and SufE positioning.

Rajleen K. Gogar, Franki Caroll, Juliana V. Conte, Mohamed Nasef, Jack A. Dunkle*, and Patrick A. Frantom*

Department of Chemistry & Biochemistry, The University of Alabama, Tuscaloosa, AL, 35487, USA

**Supporting Information (SI)**

Table S1. Kinetic data of N99A and N99D SufS variants. Values compare the kinetic data of wildtype SufS and N99 variants.^a^

| **Enzyme** | **k_cat_ (min^-1^)** | **K_SufE_ (µM)** | **K_i_ SufE (µM)** | **K_cys_ (µM)** |
| --- | --- | --- | --- | --- |
| WT SufS | 22 ± 2 | 0.8 ± 0.1 | 18 ± 3 | 63 ± 4 |
| N99A SufS | 1.5 ± 0.1^b^ | - | - | 62 ± 9 |
| N99D SufS | 1.3 ± 0.1^b^ | - | - | 57 ± 12 |

^a^Experiments performed as described in Methods section. ^b^Kinetic parameters for the N99 SufS variants determined in the absence of SufE.


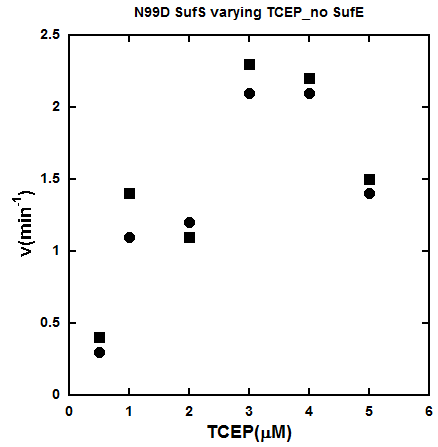


Figure S1. Kinetic analysis of N99D SufS with varying amounts of TCEP (0.5 – 5 μM), in the absence of SufE.


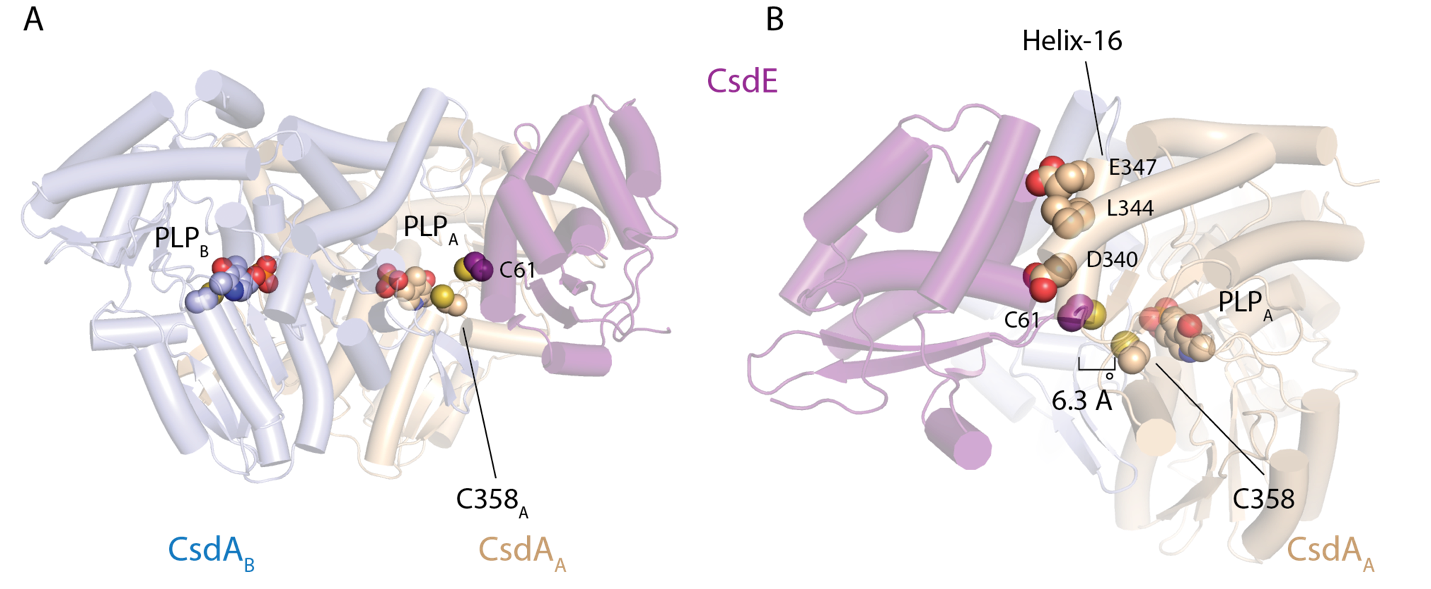


Figure S2. The crystal structure of CsdA bound to CsdE is shown in the same orientation as the SufS-SufE Rosetta model. (A) The structure given by PDB code 4lw4 is shown with the active site residues that host the persulfide, residue C358 of CsdA and C61 of CsdE, highlighted. (B) A close-up view of CsdA-CsdE interactions is shown. An induced-fit conformational change has occurred positioning the two active site cysteines much closer than in the Rosetta docked SufS-SufE model, but still slightly too far apart for persulfide transfer. Some of the residues mediating the interaction between helix-16 of CsdA and CsdE are shown.

Table S2. Primer sequences for site-directed mutagenesis to construct pET21a_SufS N99A and N99D and pET21a_SufE C51A/E107C.

| Variant | Primer | Primer Sequence (5’-3’) |
| --- | --- | --- |
| N99A SufS | Forward | GCTATTGGCGACCAGAGCGATCCCTTCCGTCGTG |
|  | Reverse | CACGACGGAAGGGATCGCTCTGGTCGCCAATAGC |
| N99D SufS | Forward | CTATTGGCGACCAGATCGATCCCTTCCGTCGTG |
|  | Reverse | CACGACGGAAGGGATCGATCTGGTCGCCAATAG |
| D346R SufS | Forward | GAGAAAACTGCCAACACGATAGGCGTGGTGTTTACCGAGA |
|  | Reverse | TCTCGGTAAACACCACGCCTATCGTGTTGGCAGTTTTCTC |
| C51A SufE | Forward | GCCCAGAGTCAGGTGTGGATTG |
|  | Reverse | GCCCTGAATGCTATTTTGTGG |
| E107C SufE | Forward | TGCAAAATGGCGCTCACCCAAC |
|  | Reverse | AAACCACGGACGCACATCG |

Figure S3. 12% SDS-PAGE of purified WT, N99A, and N99D SufS (44 kDa).

| Table S3. X-ray Data Collection and Refinement Statistics | | | |
| --- | --- | --- | --- |
|  | **N99A**  **7RUJ** | **N99D**  **7RW3** |  |
| Space group | P4_3_2_1_2 | P4_3_2_1_2 |  |
| Cell dimensions |  |  |  |
| *a, b, c*(Å) | 125.21,  125.21,  137.43 | 124.76,  124.76,  137.27 |  |
| α, β, γ (°) | 90, 90, 90 | 90, 90, 90 |  |
| Resolution (Å)^a^ | 27.12-2.50  (2.60-2.50) | 92.32-2.30  (2.36-2.30) |  |
| R_meas_ (%) | 18.0 (77.6) | 8.6 (103.2) |  |
| I/σI | 8.30 (2.0) | 22.99 (1.32) |  |
| Completeness (%) | 98.5 (96.3) | 98.8 (90.2) |  |
| Redundancy | 7.20 (5.40) | 9.91(3.75) |  |
| CC_1/2_ (%) | 98.8(78.4) | 99.9 (56.2) |  |
| Refinement |  |  |  |
| Resolution (Å) | 24.64-2.50  (2.59-2.50) | 34.20-2.30  (2.38-2.30) |  |
| No. reflections | 37,781  (3,606) | 48,152  (3,104) |  |
| R_work_/R_free_ (%) | 19.30/21.98  (26.35/33.60) | 21.63/24.03  (29.69/32.62) |  |
| No. atoms | 3,185 | 3,016 |  |
| Protein | 3,119 | 2,983 |  |
| Ligand | 16 | 15 |  |
| Water | 50 | 18 |  |
| *B* factors |  |  |  |
| Protein | 27.70 | 41.88 |  |
| Ligand | 26.78 | 36.73 |  |
| Water | 25.68 | 33.90 |  |
| R.m.s deviations |  |  |  |
| Bond lengths (Å) | 0.008 | 0.008 |  |
| Bond angles (°) | 1.00 | 0.99 |  |

^a^ Values in parentheses are for the highest-resolution shell


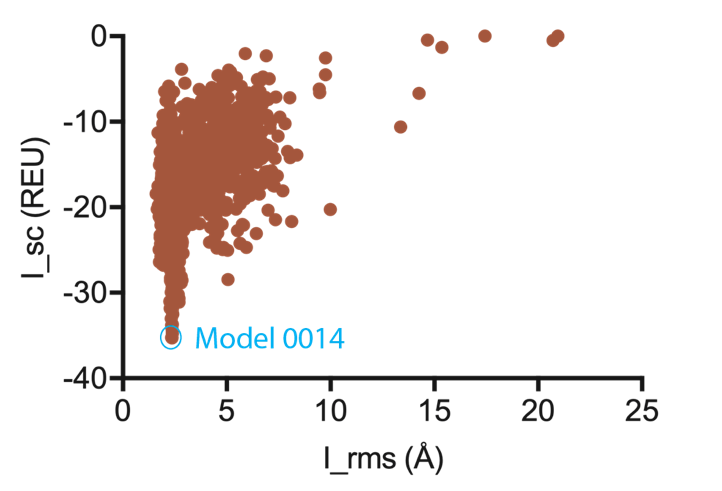


Figure S3. A plot of interface score (I_sc) in relative energy units (REU) versus interface root mean squared deviation (I_rms) for 1000 decoy structures produced by local docking within Rosetta.
